# Global Expression Analysis Revealed Novel Gender-Specific Gene Expression Features in the Blood Fluke Parasite *Schistosoma japonicum*

**DOI:** 10.1371/journal.pone.0018267

**Published:** 2011-04-06

**Authors:** Xianyu Piao, Pengfei Cai, Shuai Liu, Nan Hou, Lili Hao, Fan Yang, Heng Wang, Jianwei Wang, Qi Jin, Qijun Chen

**Affiliations:** 1 Laboratory of Parasitology, Institute of Pathogen Biology/Institute of Basic Medical Sciences, Chinese Academy of Medical Sciences and Peking Union Medical College, Beijing, China; 2 State Key Laboratory for Molecular Virology and Genetic Engineering, Institute of Pathogen Biology, Chinese Academy of Medical Sciences and Peking Union Medical College, Beijing, China; 3 Key Laboratory of Zoonosis, Ministry of Education, Institute of Zoonosis, Jilin University, Changchun, China; 4 College of Life Science and Technology, Southwest University of Nationalities, Chengdu, Sichuan, China; Universidade Federal de Minas Gerais, Brazil

## Abstract

**Background:**

*Schistosoma japonicum* is one of the remarkable Platyhelminths that are endemic in China and Southeast Asian countries. The parasite is dioecious and can reside inside the host for many years. Rapid reproduction by producing large number of eggs and count-react host anti-parasite responses are the strategies that benefit long term survival of the parasite. Praziquantel is currently the only drug that is effective against the worms. Development of novel antiparasite reagents and immune-prevention measures rely on the deciphering of parasite biology. The decoding of the genomic sequence of the parasite has made it possible to dissect the functions of genes that govern the development of the parasite. In this study, the polyadenylated transcripts from male and female *S. japonicum* were isolated for deep sequencing and the sequences were systematically analysed.

**Results:**

First, the number of genes actively expressed in the two sexes of *S. japonicum* was similar, but around 50% of genes were biased to either male or female in expression. Secondly, it was, at the first time, found that more than 50% of the coding region of the genome was transcribed from both strands. Among them, 65% of the genes had sense and their cognate antisense transcripts co-expressed, whereas 35% had inverse relationship between sense and antisense transcript abundance. Further, based on gene ontological analysis, more than 2,000 genes were functionally categorized and biological pathways that are differentially functional in male or female parasites were elucidated.

**Conclusions:**

Male and female schistosomal parasites differ in gene expression patterns, many metabolic and biological pathways have been identified in this study and genes differentially expressed in gender specific manner were presented. Importantly, more than 50% of the coding regions of the *S. japonicum* genome transcribed from both strands, antisense RNA-mediated gene regulation might play a critical role in the parasite biology.

## Introduction

Human schistosomiasis, the second only to malaria in term of morbidity and mortality, is caused by infections of *Schistosoma* species depending on the endemic region of the parasites [1]. *S. japonicum* is the causative agent of schistosomiasis perturbing millions of people in several East and Southeast Asian countries. Though schistosomal parasites are sensitive to the treatment of praziquantel, high re-infection rates in both human and animals plus the requirement of frequent administration still limit the overall success of chemotherapy. More therapeutic targets are to be defined for an optimal treatment as well as disease prevention. The recent decoding of the genome sequences of the two most pathogenic parasites, *S. mansoni* and *S. japonicum*, has paved a pivotal way for a systematic dissection of the parasite biology [2], [3], [4].

The genome of *S. japonicum* harbors in 8 pairs of chromosomes with an estimated 397 Mb containing 13,469 protein-coding sequences [3], which accounts for 4% of the genome. In the non-protein coding regions, approximately 40% is composed of repeated sequences including transposable elements (TE). Recent study indicated that the transcripts of TE could be processed into small RNAs (endogenous siRNA), which fulfilled regulatory functions from the maintenance of genome stability to stage-specific gene activation or silencing [5], [6], [7]. Genomic variation such as single nucleotide polymorphism (SNP) has been noticed but its biological significance remains to be further studied [8], [9]. The availability of the genome sequences of several schistosomal parasites plus the free-living *Schmidtea mediterranea* have paved the way for deep functional analysis on the genomes and the encoding biology of the pathogenic parasites [10], [11], [12]. Primary analyses have revealed remarked features of both parasite biology and host-parasite interaction [10], [11], [12]. Genomic sequencing project has revealed that *S. japonicum* has abandoned more than 1,000 protein coding domains as compared to the free living worm *Caenorhabditis elegans*, indicating the parasite has gained the ability to exploit host factors for its development [3]. For example, several signal transduction pathways (including those for Wnt, Notch, Hedgehog, and transforming growth factor β (TGF-β) found in human) are also present in the parasite [3]. These include endogenous hormones such as insulin, epidermal growth factor (EGF)-like and fibroblast growth factors (FGF)-like peptides. Predicted components of the Ras–Raf–MAPK and TGF-β–SMAD signaling pathways (including FGF and EGF receptors) share high sequence identity with their mammalian orthologs, indicating that schistosomes, in addition to utilizing their own signaling pathways, exploit host endocrine signals for their own development [3], [10], [13].

Schistosomal parasites are featured with very complicated developmental and biological cycles. They are the first group of organisms that are dioecious with marked differences in sexual dimorphism and biology [14], which are controlled by genetic as well as epigenetic regulation factors. Studies on stage- and gender-specific expression profiles with parasites of various developmental stages have been carried out with different methodological approaches, from manual sequencing of expression sequence tag (EST) to full-length cDNA cloning, microarray hybridization, and random sequencing [15], [16], [17], [18], [19], [20], [21]. The valuable data obtained from the genomic and post-genomic studies has facilitated tremendously in understanding parasite biology as well as parasite-host interactions (for review, see refs 10, 11, 12).

While the stage-specific transcriptomic information of *S. japonicum* keeps increasing, investigation with specific perspectives on the differences of genome-wide transcriptions of the male and female parasites has mainly been based on the availability of the genomic sequence which has been far from a complete assembly[22]. In this study, by using the high through-put RNA-seq techniques, we successfully explored the transcriptomes of male and female schistosomal parasites. The data revealed novel features of gender-specific expression and gene regulation pathways.

## Results

### Libraries of sequence tags from male and female adult worms of *S. japonicum*

**Figure 1:**
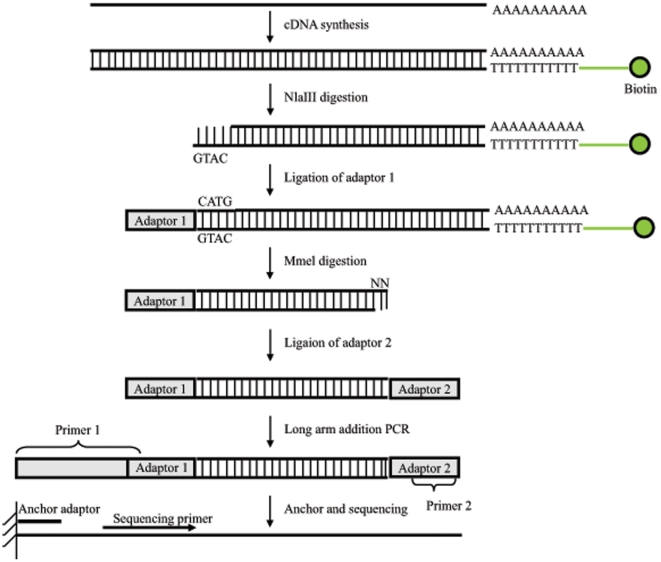
Schematic illustration of the principle and procedure of Tag preparation. Biotin-conjugated Oligo-dT was used to enrich mRNA and cDNA synthesis. The double strand cDNA was first digested with the 4 base (GTAC) recognition enzyme NlaIII, and Illumina adapter 1 was linked afterwards. Mmel was used to digest at 17 bp downstream of CATG site which was ligated with Illumina adapter 2 at the 3′ end. Sequencing anchor primers were added to the end of each fragment by PCR and the PCR product were purified and followed by Solexa sequencing.

**Figure 2:**
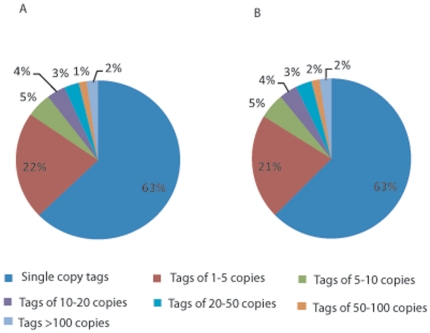
Percentage of tags in copy number identified in the two libraries (A Male worm, B Female worm). More than 60% of the tags identified in the two libraries are single copies.

In this study, we determined and compared transcriptomes of male and female adult worms of *S japonicum*. DGE (Digital Gene Expression) libraries were made, using RNA with a PolyA tail at the 3′-end of each template, for both genders, and all polyadenylated RNA was sequenced using Solexa (Illumina) high through-put technology (Figure 1). The two libraries (male and female adult worms) contained 3,705,287 and 3,672,014 unfiltered tags. After removal of tags containing ambiguous base calls and adaptor tags, there were 3,660,835 (male) and 3,693,835 (female) clean tags and the number of distinct tags in the two libraries of male and female was 219,628 and 213,310, respectively (Table 1). The clean tags were mapped onto the *S. japonicum* genome of SGST (http://lifecenter.sgst.cn) and the relationship between sequence tags and genes was then built up. For genes with multi-tags, the total distinct expressed tags were taken into account as the gene expression value. Most of the tags were from highly expressed genes (Figure 2 and Table S1). The redundancy for Sjc-F and Sjc-M was respectively 94.2% and 94.1% which indicated the sequencing quantity should be enough for both libraries (Table 1). Of the 360,955 unique tags, 71,983 can be found in both libraries. Male and female specific tags accounted for 3.85% and 4.35% respectively. The number of clean distinct tags was 141,327 and 147,645 in Sjc-F and Sjc-M, respectively (Table 1). As shown in figure 2, the most abundant tags (63%) were single copy and tags with more than 10 copies accounted only around 3% in both female and male worms (Figure 2). All sequence data has been deposited in the database (http://www.ncbi.nlm.nih.gov/geo/info/faq.html#seq) with an accession number of GSE26845.

**Table 1:** Expression profiles of sequence reads in the two libraries.

	Sjc-F	Sjc-M
Distinct clean reads	213,310	219,628
Sex-specific reads	141,327	147,645
Matched to genome	57,395	55,498
Redundancy (%)	94.2	94.1

Distinct reads represent the number of distinct sequence reads in the two libraries, Sjc-F and Sjc-M. Sex-specific reads represent number of sequence reads specific to female (Sjc-F) or male (Sjc-M) parasite. The numbers of the distinct reads from the two libraries that matched to the genomic sequences were listed. The redundancy of the two libraries was calculated according to the formula (Redundancy = 100-(Total Clean Distinct Tags/Total Tags x 100).

### Genes differentially expressed in male and female parasites

**Figure 3:**
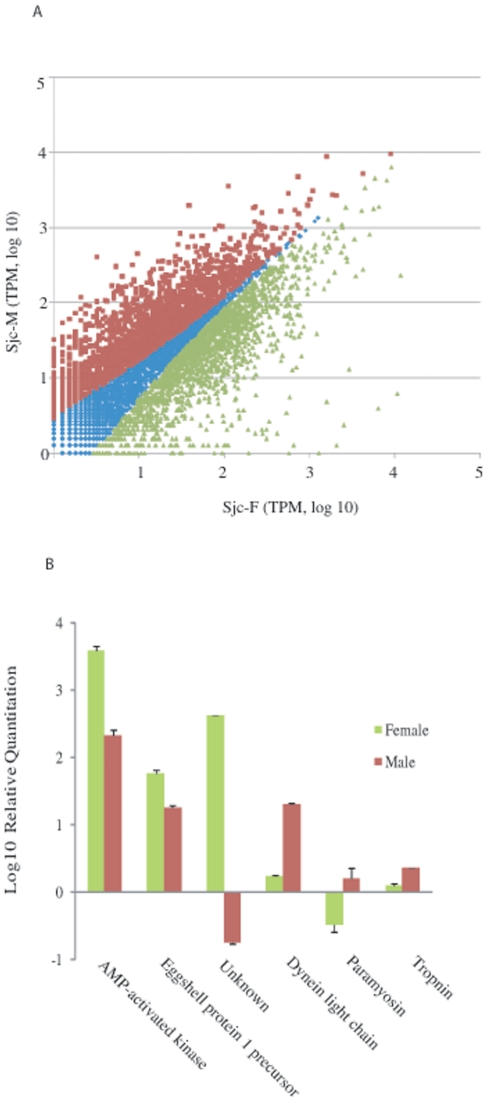
Tags represented differential expression in male and female parasites. **A** Distribution by Scatter plotting of expressed sequence tags identified in male and female parasites. Tags biased towards male parasite were in red color, while tags biased towards female parasite were labeled in green color. **B** Verification of gender-biased expression of 6 genes by real-time RT PCR. The differences in copy numbers of transcripts relative to that of α-tubulin were presented in log 10 scale.

Tags that could specifically match to the reference genes of *S japonicum* generated expression data of 9,239 genes, accounted for 73% of genes in the annotated genome which was estimated to have 13,469 genes in the genome [3]. A total of 4,732 (35%) distinct genes were found differentially expressed between male and female, of which 2,545 genes up-regulated and 2,187 genes down-regulated in male versus female adult worms (Figure 3A and Table S2). Genes showed significant differences in expression were those coding proteins with functions associated with biological process, cellular component or molecular functions (Table S2). Genes related to the function of genetic information processing which was more biased to the female parasite, while genes with function related to interaction with host (environmental information processing) were more active in the male parasites. To evaluate whether the number of sequencing tags that could reflect the patterns of differentially expressed genes between male and female parasites, transcripts of 6 genes of AMP-activated kinase, eggshell protein 1 precursor, an unknown gene (Sjc_0024870), dynein light chain, paramyosin, and tropnin were analyzed by quantitative PCR. The results from quantitative PCR correlated with the number of sequence tags that were significantly different between male and female parasite (Figure 3B).

### Half of the coding regions in the genome of *S. japonicum* was transcribed from both strands

**Figure 4:**
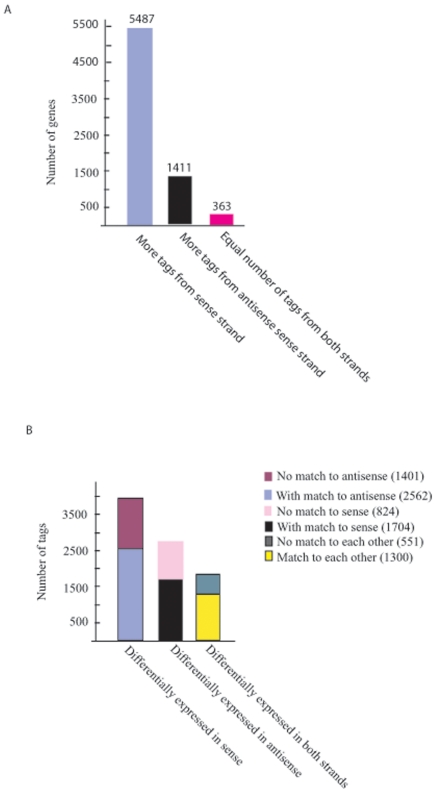
Sequence tags identified from both sense and antisense strands of the genome. **A** Gene numbers that with differential transcription patterns of the two DNA strands. Genes with more transcription from the sense strand were dominant. **B** Tags differentially expressed in male and female parasites.

When mapping the sequence tags to the genome we found that, of the genes (9,239) with unambiguous tags detected, 7,261 genes have tags transcribed from both sense and antisense strands. Thus nearly 50% of the genes annotated in the genome of *S. japonicum* were found transcribed from both strands. Of these genes, 5,487 genes had tags corresponding to sense strands more than that from antisense strands, and 1411 genes had more tags from the antisense strands than that from the sense strands. While 363 genes have equal number of tags generated from both strands (Figure 4A, Table S3).

Further comparative analysis on the sequence tags between male and female parasites revealed that 3,963 tags from sense strand were significantly different in copy number between male and female parasites. Of which, 2,562 tags had antisense and their cognate sense transcripts co-expressed (higher levels of sense tags also yield higher antisense tags counts, Figure 4B), 1,401 tags had no matched antisense tags. There were 2,528 antisense tags which were differentially expressed in the two sexes of the parasite, of which 1,704 had sense counterparts co-expressed and 824 was discordant with the sense strand. 1,851 genes had differentially expressed tags from both sense and antisense strands, with 1,300 tags were co-expressed, and 551 tags were discordant (Figure 4B).

### Identification of different biological or metabolic pathways between male and female parasites

**Figure 5:**
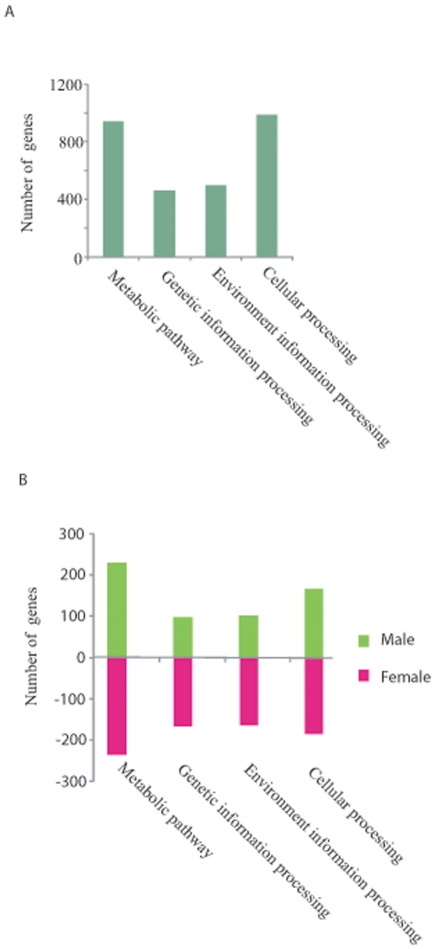
Functional categorization of genes identified in male and female parasites. **A** Number of genes that can be categorized into four main functional groups (Metabolic pathway, genetic information processing, environmental information processing and cellular processing). **B** Number of genes within the four functional categories that showed up- or down-regulation in male parasite compared to female counterpart.

Gene categorization based on potential functions of the coded proteins was performed. Sequence tags from 2,148 genes can be categorized into different functions or biological pathways (Figure 5, Table 2, and Table S4). Of which, 940 genes related to metabolic pathways, 475 genes were with functions related to genetic information processing, 495 genes were related to responses to environmental changes, and 958 genes were related to cellular processing (Figure 5A).

**Table 2:** Number of genes potentially involved in biological pathways and differentially expressed in male and female parasites.

Pathways	Total genes identified		Genes up-regulated(P<0.05)	
	Sjc-M	Sjc-F	Sjc-M	Sjc-F
**Metabolism**				
Amino acids	134	134	48	43
Biosynthesis of secondary metabolites	14	15	6	7
Carbohydrate	102	97	27	37
Energy	79	72	19	26
Glycan biosythesis	50	49	13	14
Lipid	50	49	19	10
Cofactors and vitamins	45	43	11	20
Nucleotides	41	39	17	9
Xenobiotics biodegradation	20	17	7	5
**Genetic information processing**				
Replication and repair	81	75	11	30
Transcription	45	43	9	18
Translation	144	144	28	62
Folding, sorting and degradation	119	119	42	47
**Environmental information processing**				
Membrane transport	28	30	12	5
Signal transduction	160	159	53	32
Signaling molecules and interaction	25	28	10	4
**Cellular processes**				
Cell communication	108	102	35	20
Cell growth and death	85	76	13	35
Cell motility	43	41	13	7
Development	31	31	14	2
Endocrine system	100	97	17	31
Immune system	72	75	23	15
Nervous system	44	43	8	11
Sensory system	17	12	1	3
**Others**	255	252	74	77

Genes with differential expression patterns between male and female parasites were also identified (Table S5, S6), of the 940 genes with functions associated to metabolism, 230 genes were up-regulated and 238 genes are down-regulated in male compared to female parasites. Of the 475 genes with functions related to genetic information processing, 98 genes were up-regulated and 168 genes were down-regulated in the male parasite. 102 genes related to environmental information processing were up-regulated and 65 genes were down regulated in male parasites. 168 genes function in cellular processing were more active in male parasites, while 185 genes were more silent than female counterpart (Figure 5B). Among the metabolic pathways identified in the parasites, the expression of 5 genes related to the xenobiotic metabolism was found up-regulated in female parasites (Table S5, S6).

## Discussion

The draft genomic sequence of *S. japonicum* has been available [3], but functional determination of genes related to important biological significance will likely rely on the analysis of mRNA transcripts and the encoded proteins, since the multi-cellular nature of the pathogen and its specific structure of tegument has made it difficult to carry out genetic manipulation directly on the parasite [21]. In this study, by combining the powerful Digital Gene Expression (DGE)-tag and high through-put RNA-seq technique [23], the global transcriptomes of male and female *S. japonicum* were obtained and compared. DGE offers distinct advantages over other methods (such as array-based gene-expression analysis systems) for transcriptomic studies. First, it has a better coverage and an ability to measure low-abundance genes, find unknown transcripts with minimal background noise for increased sensitivity. Secondly, as demonstrated in Figure 1, all sequence tags were anchored on a chip matrix at the 3′ side before sequencing, thus only the cDNA strand (complimentary to the polyA-tailed RNA template) was sequenced. The advantages of this approach are that most adenylated transcripts can be obtained and the step of cDNA cloning is not needed. Further, the rationale in tag preparation was that the restriction enzyme (NlaIII) would cleave at the 3′ most CATG site, thus the 3′ UTR (Un-translating region) information will be critical for the following tag annotation. To avoid false positive of CATG site, we used 3 kb as the cutoff value to define the 3′ UTR of the selected RNA templates. The CATG cleavage sites were identified in the gene accompanied with 3 kb potential 3′ UTR using in-house perl script. Thus, contrast to normal EST sequencing which mainly obtains sequence information close to the 5′ end of the templates, the DGE method explored here could target the mRNA sequences which were more likely in full-length. Though deep (or random) sequencing can generate genome-wide transcriptome information, it does not discriminate strand-specific transcription. Further, all sequence tags were mapped to the protein-coding genes with non-coding sequences dismissed, thus small transcripts such as pre-microRNAs and transcripts from non-coding regions were not included in the analysis.

The number of sequence tags identified in male and female parasites was similar (Figure 2 and Table S1). However, around one third of genes in the genome were found with bias in preferential expression between male and female. Interestingly, the number of genes with preferential expression in male and female parasite was similar (Figure 3A and Table S2). The differences in gene expression between male and female parasites were related to the function of genetic information processing which was more biased to the female parasite, which was likely due to the production of eggs. While genes with function related to interaction with host (environmental information processing) were more active in the male parasites, this was presumably due to the physiological character of male parasite which was much larger than the female and most of its surface was exposed to the host while female parasite was held in the cavity of the male. Further, previous studies with microarray identified around 1,000 genes that were differentially expressed in either male or female parasite [19], [24]. The reason that low numbers of genes identified in early studies was likely due to the in-availability of a complete genome sequence when the studies were performed. The advantage of the current study is that the readout does not depend on the genome sequence. Thus the number of genes identified with differential expression in male and female parasite was more than that with other approaches [19], [22], [24].

Gender-specific transcriptome analysis revealed that more than 2,000 genes were potentially involved in metabolic pathways or biological functions (Figure 5 and **Table S4**). Among the metabolic pathways identified in the parasites, the expression of genes related to the xenobiotic metabolism was found more interesting. Xenobiotic metabolism reactions often function in detoxifying poisonous compounds [25]. The reactions contain three phases. In phase I, enzymes such as cytochrome P450 oxidases introduce reactive or polar groups into xenobiotics. These modified compounds are then conjugated to polar compounds in phase II reactions. These reactions are catalyzed by transferase enzymes such as glutathione S-transferases (GST). In phase III, the conjugated xenobiotics are recognized by efflux transporters and pumped out of cells [25]. Proteins encoded by these genes are likely involved in fertilization or egg production in the female parasite. Studies on *S. mansoni* has reported functions of P450 and GST in the parasite [26]. However, this is the first report which reveals more complete connection of the enzymes in the xenobiotic metabolism pathway in *S. japonicum*. So far, GST has been regarded as a best candidate for development of anti-fecundity vaccine for japonicum schistosomiasis [27]. In light of the components identified in the pathways related to the reproduction of the parasite, more molecules such as P450 homologue might be potential candidate in the vaccine development. Further, genes with functions related to the pairing of the two sexes were found differentially expressed. Male parasite expressed more genes related to WNT (originally been identified as a recessive mutation affecting wing and haltere development in *Drosophila melanogaster*) signaling pathway which might be beneficial for embryo development in female parasites. Interestingly, genes encoded actin proteins were found more active in female parasites than male parasites, whether this related to the egg-shedding function or the pairing of the two sexes remains further elucidation. Furthermore, the axon guidance pathway was found more active in female than male. Compounds targeting these pathways may effectively block parasite development and reduce pathological reaction in the liver of the host.

The discovery of tremendous antisense transcripts from the coding region is remarkable. The estimated number of protein-coding genes in the *S. japonicum* genome is 13,469, while 7,261 genes were found transcribed from both strands. To our knowledge, this is the first observation in *S. japonicum* that more than 50% of the protein-coding genes were bi-directionally transcribed. It much be pointed out that previous studies in *S. mansoni* using a microarray already found bi-directional transcription in 7% of the active “no match” genes [28]. Thus bi-directional transcription is likely a common feature in schistosomal parasites. Though most of the transcripts were from sense strands of the genes, more than 1,000 genes were found to have more antisense than sense transcripts and around 500 genes were transcribed symmetrically. Since the RNA templates were selected based on the poly-A tail, thus the antisense transcripts were likely polyadenylated. It cannot be ruled out that some of the anti-sense RNAs may encode proteins, but it is unlikely that all polyadenylated antisense RNAs do so. Recent study on the antisense transcripts in human found that the pseudogenes could be sources of natural antisense transcripts [29]. Transcripts from pseudogenes form hybrids with that of parental genes, which will be further processed into regulatory endogenous siRNAs. Though it could not be ruled out that such a mechanism also existed in *S. japonicum*, it is unlikely that the parasite harbors so many pseudogenes in the genome, as antisense transcripts complementary to more than half of the protein-coding genes were detected. Thus, some of the antisense transcripts must be a result of bi-directional transcription, at least in the adult worms. The mechanism behind the bi-directional transcription is still not known; but, with the discovery of NAT (natural antisense transcripts)-derived endogenous siRNAs in the parasite [5], it can be hypothesized that some, if not all, sense and antisense RNA hybrids are the sources of NAT-derived endo-siRNAs [7]. However, it is also possible that some of the antisense transcripts exerted post-transcriptional regulation through direct hybridization with the mRNA templates. Nevertheless, the finding in this study has opened up new avenue for dissection of parasite biology regarding the function of antisense RNA-dependent gene regulation.

In this study, transcripts of 73% of the genes in *S. japonicum* genome was identified by high-through-put sequencing, of which, 35% (4,732/13,469) was preferential expressed in either male or female parasite. More than 900 genes involved in metabolic and biological pathways were identified and genes that were differentially expressed in gender specific manner were analyzed. Further, polyadenylated antisense RNAs were mapped to more than 50% of the coding regions in *S. japonicum* genome, indicating bi-directional transcription were common, at least in adult worm stage of the parasite. Antisense-mediated gene regulation might play a critical role in the parasite biology.

## Methods

### Parasites and RNA purification

*S. japonicum*-infected snails were collected from the endemic area in Jiangxi province. Cercarie were released from the snails in room temperature (around 25 degree) under a lamp. One New Zealand white female rabbit (5 month old) was infected with 1500-2000 cercarie for 42 days. Mature adult parasites were harvested from the infected rabbit by flushing the blood vessels with PBS as described earlier [5], [30]. Male and female parasites were manually separated and total RNA from the parasites was purified with Trizol reagent (Invitrogen, CA, USA) as described [5], [30].

### Generation of expression tags of male and female parasites for sequencing

Messenger RNA from male and female *S. japonicum* parasite was selectively purified from total RNA using oligo-(dT) conjugated magnetic beads (Dynabeads®, Invitrogen). Complementary DNA (cDNA) was synthesized guided by oligo-(dT) as a primer. Sequencing tags were generated as illustrated in Fig. 1. Briefly, double stranded cDNA sample was digested with the endonuclease NlaIII that recognizes the CATG sites on cDNAs. After cleavage, the 3′-regions of the cDNAs attached on the magnetic beads were selected. The first sequencing adapter (Illumina adapter 1) [31] was added to the 5′ ends of each fragment which was further digested with MmeI, an enzyme cuts 17 bp downstream of the CATG site. After removing 3′ fragments with magnetic beads precipitation, Illumina adapter 2 was introduced at 3′ ends of the tags to generate tag library with different adapters at both ends. The fragments were PCR amplified and the 85 base strips were purified by 6% TBE PAGE Gel electrophoresis and sequenced with the Solexa high-throughput sequencing technology. The advantage of this approach is that transcripts from both strands (sense and anti-sense) can be targeted and sequenced.

### Sequence analysis

After removing the low quality and adaptor tags, the clean sequence tags were mapped onto the gene reference tag data set and the relationship between sequence tags and genes were then built up. For genes with multi-tags, the total distinct expressed tags were taken into account as the gene expression value. For tags that mapped to different genes, the mean value of tag number was used as the expression level for each gene.

Reads with CATG site were selected and mapped to the genome sequences. Sequences that with complete match to the genome sequences were further analyzed for differential expression. We employed IDEG6 (http://telethon.bio.unipd.it/bioinfo/IDEG6/) to identify differentially expressed mRNAs based on their relative abundance which was reflected by total count of individual sequence read between the two libraries. The general Chi test was employed which has been proved to be one of the most efficient tests [32]. Finally, genes with a P value < =  0.05 were deemed to be significantly different between the two libraries.

Gene sequences were firstly blasted with Kyoto Encyclopedia of Genes and Genomes database (KEGG, release 50) (http://nematode.net/cgi-bin/keggview.cgi and http://www.nematode.net/FTP/index.php) with E values < =  1e-10 [33]. The KO information was retrieved from blast result using which the possible pathway information for each gene could be identified. Domain information was annotated by InterProScan and functional assignments were mapped onto Gene Ontology (GO). WEGO was employed to do GO classification and draw GO tree [34].

### Verification of gender-specific transcripts by real-time quantitative RT-PCR

Total RNA of *S. japonicum* (adult male and female worms) was extracted using Trizol reagent (Invitrogen, CA, USA). The RNAs were dissolved in diethylpyrocarbonate (DEPC)-treated water and reverse transcribed with 200 U SuperScript™ III Reverse Transcriptase (Invitrogen) according to the manufacturer's instruction. The following primers were designed as forward and reverse primers based on the female, male specific tags and α-tubulin gene (endogenous control): AMP-activated kinase F: 5′-TGCTAGTGGTAAATGGGGTGT-3′, R: 5′-TTCATTGTACCATTGGATATTTTCAT-3′. Eggshell protein 1 precursor F: 5′-TGGTGGTAAGAATGGTGGTG-3′, R: 5′-CACACATTACGATATTACAGTGAGATG-3′. Unknown (Sjc_0024870*: S. japonicum* expressed protein, putative mRNA) F: 5′-CACGACATCAACATGAGGGTA-3′, R: 5′-ACCCGAATATCGTGAAACAGA-3′. Dynein light chain F: 5′-GCTGCAATGGCTATGGATAAA-3′, R: 5′-TCCACGATCTTCCAGTGAGA-3′. Paramyosin F: 5′-CTCAAAGCAGCCATAACA-3′, R: 5′-TCTCCTCCTCCAACTGAA-3′. Tropnin F: 5′-CGATGGAAAGTCTGAAGC-3′, R: 5′-ACGTTCCCCTCTACGAAA-3′. α-tubulin F: 5′-CATGGTAGACAACGAAGCTATTTATGA-3′, R: 5′-GATTAGTGTAGGTTGGACGCTCTATG-3′.

We used α-tubulin transcript as the endogenous control. Quantitative RT-PCR was conducted in triplicate and each reaction underwent 40 amplification cycles using an Applied Biosystems 7300 real-time PCR system (Applied Biosystems, Foster City, USA) with cDNA equivalent to 15 ng of total RNA, 200 mM of primers and 12.5 µl SYBR Green PCR Master Mix (ABI, USA) adjusted to final volume of 25 µl with DEPC-treated water. Dissociation curves were generated for each sample to verify the amplification of a single PCR product. The Relative expression was analyzed using the SDS 1.4 software (Applied Biosystems, Foster City, USA). Due to the fact that the transcription of α-tubulin gene in male was 2 times higher than in female[19], a step of normalization was included in the final analysis.

## Supporting Information

Table S1 Description of the libraries generated with sequence tags from male and female *S. japonicum*. The first column (Class) defined the sequence classes. In the columns of Sjc-F and Sjc-M, # represents the number of tags; % represents the percentage of clean tags with different copy numbers in the total clean tag pools of female and male parasite respectively.
(DOC)

Table S2 Genes showed significant differences in expression in female versus male parasite and the biological functions associated. The function of genes identified were classified into general (First Class) and more defined (Second Class). The number of genes up- (# of Up), down-regulated (# of Down) as well as the contig names were listed.
(XLS)

Table S3 Tags mapped to either sense, antisense strand or both stands of the genes identified. The first column is the gene name, the second column ‘Both’ means gene expressed in both strand. ‘# of Detected’ is the number of tags detected by sequencing. ‘Total Express’ means total times of detected tags including both female and male. ‘Sjc-F Expression and Sjc-M Expression’ means total times of detected tags in female and male respectively. ‘Total TPM’ means total times of detected tags per million, and Sjc-F TPM, Sjc-M TPM means total times of detected tags per million in female and male respectively. ‘M-F’ means difference between the TPM value of Sjc-M and Sjc-F. Up in the Mark column means the TPM value of Sjc-M is higher than that of Sjc-F. The last column ‘Tags’ represents tag positions in genome, for example. “Y” means the tag is distinct. The numbers represent the position of the “CATG” from the 3′ end of the gene, total TPM and the TPMs of the same tag in Sjc-F and Sjc-F respectively.
(XLS)

Table S4 Tags mapped to genes involved in metabolic and other biological functions. The first column lists the metabolic pathways and classified biological functions identified. The second column represents the number of genes involved and the thirst column represents the contig names.
(XLS)

Table S5 Genes involved in metabolic and other biological functions which were up-regulated in male parasites.
(XLS)

Table S6 Genes involved in metabolic and other biological functions which were down-regulated in male parasites.
(XLS)

## References

[1] King CH, Dickman K, Tisch DJ (2005). Reassessment of the cost of chronic helmintic infection: a
meta-analysis of disability-related outcomes in endemic
schistosomiasis.. Lancet.

[2] Webster JP, Oliviera G, Rollinson D, Gower CM (2010). Schistosome genomes: a wealth of information.. Trends Parasitol.

[3] (2009). The *Schistosoma japonicum* genome reveals
features of host-parasite interplay.. Nature.

[4] Berriman M, Haas BJ, LoVerde PT, Wilson RA, Dillon GP (2009). The genome of the blood fluke *Schistosoma
mansoni*.. Nature.

[5] Hao L, Cai P, Jiang N, Wang H, Chen Q (2010). Identification and characterization of microRNAs and endogenous
siRNAs in *Schistosoma japonicum*.. BMC Genomics.

[6] Tam OH, Aravin AA, Stein P, Girard A, Murchison EP (2008). Pseudogene-derived small interfering RNAs regulate gene
expression in mouse oocytes.. Nature.

[7] Carthew RW, Sontheimer EJ (2009). Origins and Mechanisms of miRNAs and siRNAs.. Cell.

[8] Wang TP, Shrivastava J, Johansen MV, Zhang SQ, Wang FF (2006). Does multiple hosts mean multiple parasites? Population genetic
structure of *Schistosoma japonicum* between definitive host
species.. Int J Parasitol.

[9] Rudge JW, Lu DB, Fang GR, Wang TP, Basanez MG (2009). Parasite genetic differentiation by habitat type and host
species: molecular epidemiology of *Schistosoma japonicum* in
hilly and marshland areas of Anhui Province, China.. Mol Ecol.

[10] Han ZG, Brindley PJ, Wang SY, Chen Z (2009). *Schistosoma* genomics: new perspectives on
schistosome biology and host-parasite interaction.. Annu Rev Genomics Hum Genet.

[11] Brindley PJ, Mitreva M, Ghedin E, Lustigman S (2009). Helminth genomics: The implications for human
health.. PLoS Negl Trop Dis.

[12] Chuan J, Feng Z, Brindley PJ, McManus DP, Han Z (2010). Our wormy world genomics, proteomics and transcriptomics in East
and southeast Asia.. Adv Parasitol.

[13] You H, Zhang W, Jones MK, Gobert GN, Mulvenna J (2010). Cloning and characterisation of *Schistosoma
japonicum* insulin receptors.. PLoS One.

[14] Hirai H, Taguchi T, Saitoh Y, Kawanaka M, Sugiyama H (2000). Chromosomal differentiation of the *Schistosoma
japonicum* complex.. Int J Parasitol.

[15] Fan J, Minchella DJ, Day SR, McManus DP, Tiu WU (1998). Generation, identification, and evaluation of expressed sequence
tags from different developmental stages of the Asian blood fluke
*Schistosoma japonicum*.. Biochem Biophys Res Commun.

[16] Fung MC, Lau MT, Chen XG (2002). Expressed sequence tag (EST) analysis of a *Schistosoma
japonicum* cercariae cDNA library.. Acta Trop.

[17] Hu W, Yan Q, Shen DK, Liu F, Zhu ZD (2003). Evolutionary and biomedical implications of a *Schistosoma
japonicum* complementary DNA resource.. Nat Genet.

[18] Gobert GN, McInnes R, Moertel L, Nelson C, Jones MK (2006). Transcriptomics tool for the human *Schistosoma*
blood flukes using microarray gene expression profiling.. Exp Parasitol.

[19] Moertel L, McManus DP, Piva TJ, Young L, McInnes RL (2006). Oligonucleotide microarray analysis of strain- and
gender-associated gene expression in the human blood fluke,
*Schistosoma japonicum*.. Mol Cell Probes.

[20] Liu F, Lu J, Hu W, Wang SY, Cui SJ (2006). New perspectives on host-parasite interplay by comparative
transcriptomic and proteomic analyses of *Schistosoma
japonicum*.. PLoS Pathog.

[21] Brindley PJ, Pearce EJ (2007). Genetic manipulation of schistosomes.. Int J Parasitol.

[22] Gobert GN, Moertel L, Brindley PJ, McManus DP (2009). Developmental gene expression profiles of the human pathogen
*Schistosoma japonicum*.. BMC Genomics.

[23] Saha S, Sparks AB, Rago C, Akmaev V, Wang CJ (2002). Using the transcriptome to annotate the genome.. Nat Biotechnol.

[24] Fitzpatrick JM, Johansen MV, Johnston DA, Dunne DW, Hoffmann KF (2004). Gender-associated gene expression in two related strains of
*Schistosoma japonicum*.. Mol Biochem Parasitol.

[25] Murphy PJ (2001). Xenobiotic metabolism: a look from the past to the
future.. Drug Metab Dispos.

[26] Vande Waa EA, Campbell CK, O'Leary KA, Tracy JW (1993). Induction of *Schistosoma mansoni* glutathione
S-transferase by xenobiotics.. Arch Biochem Biophys.

[27] Shuxian L, Yongkang H, Guangchen S, Xing-song L, Yuxin X (1997). Anti-fecundity immunity to *Schistosoma japonicum*
induced in Chinese water buffaloes (Bos buffelus) after vaccination with
recombinant 26 kDa glutathione-S-transferase (reSjc26GST).. Vet Parasitol.

[28] Verjovski-Almeida S, Venancio TM, Oliveira KC, Almeida GT, DeMarco R (2007). Use of a 44k oligoarray to explore the transcriptome of
*Schistosoma mansoni* adult worms.. Exp Parasitol.

[29] Muro EM, Andrade-Navarro MA (2010). Pseudogenes as an alternative source of natural antisense
transcripts.. BMC Evol Biol.

[30] Cai P, Bu L, Wang J, Wang Z, Zhong X (2008). Molecular characterization of *Schistosoma
japonicum* tegument protein tetraspanin-2: sequence variation
and possible implications for immune evasion.. Biochem Biophys Res Commun.

[31] Bennett S (2004). Solexa Ltd.. Pharmacogenomics.

[32] Gu X, Lee JJ (2010). A simulation study for comparing testing statistics in
response-adaptive randomization.. BMC Med Res Methodol.

[33] Wylie T, Martin J, Abubucker S, Yin Y, Messina D (2008). NemaPath: online exploration of KEGG-based metabolic pathways for
nematodes.. BMC Genomics.

[34] Ye J, Fang L, Zheng H, Zhang Y, Chen J (2006). WEGO: a web tool for plotting GO annotations.. Nucleic Acids Res.

