# Supplementary material for: Global Expression Analysis Revealed Novel Gender-Specific Gene Expression Features in the Blood Fluke Parasite Schistosoma japonicum
Source: PLoS One. 2011 Apr 6;6(4):e18267. doi: 10.1371/journal.pone.0018267 (PMC3071802; doi:10.1371/journal.pone.0018267)
Supplement: Table S1 — Description of the libraries generated with sequence tags from male and female S. japonicum. The first column (Class) defined the sequence classes. In the columns of Sjc-F and Sjc-M, # represents the number of tags; % represents the percentage of clean tags with different copy numbers in the total clean tag pools of female and male parasite respectively. (DOC) [file pone.0018267.s001.doc]

| Class | Sjc-F | | | | Sjc-M | | | |
| --- | --- | --- | --- | --- | --- | --- | --- | --- |
|  | Distinct Tag | | Total Tag | | Distinct Tag | | Total Tag | |
|  | # | % | # | % | # | % | # | % |
| Raw Data | 219406 |  | 3672014 |  | 226267 |  | 3705287 |  |
| Low Quality TAG (containing Ns) | 6095 |  | 11065 |  | 6638 |  | 11412 |  |
| Adaptor Tags | 1 |  | 114 |  | 1 |  | 40 |  |
| Clean Tags | 213310 |  | 3660835 |  | 219628 |  | 3693835 |  |
| Copy Num =1 | 133888 | 62.77 | 133888 | 3.66 | 137318 | 62.52 | 137318 | 3.72 |
| Copy Num >1 | 79422 | 37.23 | 3526947 | 96.34 | 82310 | 37.48 | 3556517 | 96.28 |
| Copy Num >5 | 32499 | 15.24 | 3395482 | 92.75 | 35635 | 16.23 | 3425417 | 92.73 |
| Copy Num >10 | 21782 | 10.21 | 3314413 | 90.54 | 24170 | 11.00 | 3338487 | 90.38 |
| Copy Num >20 | 14152 | 6.63 | 3202932 | 87.49 | 16165 | 7.36 | 3221181 | 87.20 |
| Copy Num >50 | 7881 | 3.69 | 3002643 | 82.02 | 8956 | 4.08 | 2988888 | 80.92 |
| Copy Num >100 | 4754 | 2.23 | 2781093 | 75.97 | 5363 | 2.44 | 2731907 | 73.96 |
